# The presenilin 1 mutation P436S causes familial Alzheimer's disease with elevated Aβ43 and atypical clinical manifestations

**DOI:** 10.1002/alz.13904

**Published:** 2024-06-02

**Authors:** Charles Arber, Christopher R. S. Belder, Filip Tomczuk, Rebecca Gabriele, Yazead Buhidma, Clíona Farrell, Antoinette O'Connor, Helen Rice, Tammaryn Lashley, Nick C. Fox, Natalie S. Ryan, Selina Wray

**Affiliations:** ^1^ Department of Neurodegenerative Disease UCL Queen Square Institute of Neurology London UK; ^2^ Dementia Research Centre UCL Queen Square Institute of Neurology London UK; ^3^ UCL Queen Square Institute of Neurology UK Dementia Research Institute at UCL London UK; ^4^ Adelaide Medical School The University of Adelaide Adelaide South Australia Australia; ^5^ Department of Genetics Institute of Psychiatry and Neurology Warsaw Poland

**Keywords:** AD, Aβ, familial Alzheimer's disease, iPSC, PALP, PSEN1

## Abstract

**INTRODUCTION:**

Familial Alzheimer's disease (fAD) is heterogeneous in terms of age at onset and clinical presentation. A greater understanding of the pathogenicity of fAD variants and how these contribute to heterogeneity will enhance our understanding of the mechanisms of AD more widely.

**METHODS:**

To determine the pathogenicity of the unclassified *PSEN1* P436S mutation, we studied an expanded kindred of eight affected individuals, with magnetic resonance imaging (MRI) (two individuals), patient‐derived induced pluripotent stem cell (iPSC) models (two donors), and post‐mortem histology (one donor).

**RESULTS:**

An autosomal dominant pattern of inheritance of fAD was seen, with an average age at symptom onset of 46 years and atypical features. iPSC models and post‐mortem tissue supported high production of amyloid beta 43 (Aβ43). PSEN1 peptide maturation was unimpaired.

**DISCUSSION:**

We confirm that the P436S mutation in *PSEN1* causes atypical fAD. The location of the mutation in the critical PSEN1 proline‐alanine‐leucine‐proline (PALP) motif may explain the early age at onset despite appropriate protein maturation.

**Highlights:**

- *PSEN1* P436S mutations cause familial Alzheimer's disease.
- This mutation is associated with atypical clinical presentation.
- Induced pluripotent stem cells (iPSCs) and post‐mortem studies support increased amyloid beta (Aβ43) production.
- Early age at onset highlights the importance of the PALP motif in PSEN1 function.

## 1 BACKGROUND

Familial Alzheimer's disease (fAD) is caused by autosomal dominantly inherited mutations in the amyloid precursor protein (APP) and presenilin 1 or 2 (*PSEN1/2*) genes.^1^, ^2^, ^3^ The normal cleavage of APP by PSEN1/2 (the catalytic subunit of γ‐secretase) is disrupted by fAD‐associated mutations, leading to changes in amyloid beta (Aβ) production, and predisposing the deposition of Aβ into amyloid plaques. Plaques composed of Aβ are characteristic of AD, and Aβ is thought to initiate a disease cascade.^4^

Biochemically, fAD mutations can lead to either (1) increased overall production of Aβ or (2) elevated production of longer, more aggregation prone species such as Aβ42 and Aβ43 in relation to shorter species such as Aβ40 and Aβ38.^5^ Aβ has been shown to be cleaved by γ‐secretase in two major carboxypeptidase cleavage pathways: Aβ49 > 46 > 43 > 40 and Aβ48 > 45 > 42 > 38.^6^, ^7^ Mutations in *PSEN1* destabilize the Aβ to γ‐secretase complex, thereby releasing longer forms of Aβ prior to the final processing step.^8^

More than 300 mutations in *PSEN1* have been described (alzforum.org). Mutations are distributed along the entire PSEN1 peptide and commonly cause single amino acid substitutions. Different mutations display remarkable heterogeneity in (1) age at symptom onset, which can range from 24 to over 65 years of age^9^, ^10^; and (2) clinical presentation: most mutations present with cognitive decline, whereas others may present with additional motor features, such as spastic paraparesis, which can precede cognitive decline.^9^, ^11^

Induced pluripotent stem cells (iPSCs) offer a unique opportunity to study patient‐derived neurons expressing physiological doses of mutant proteins in the cell type affected by disease.^12^ Our previous work has shown that different mutations in *PSEN1* can have distinct effects on APP processing and Aβ production, with PSEN1 protein stability and maturity affected by different mutations.^13^ It is notable that alterations in Aβ processing are conserved between iPSC models and patient plasma,^14^ validating the relevance of iPSCs to model Aβ production in patients.

Aβ43 is a particularly aggregation‐prone species,^15^ which is commonly overlooked. Employing iPSC models, work by us and by others has demonstrated that mutations in *PSEN1*, such as R278I, E280G, and L435F, lead to an increased relative production of Aβ43 compared to shorter peptides.^13^, ^16^, ^17^, ^18^ These mutations frequently have atypical clinical presentations, with prominent motor features including spastic paraparesis or extrapyramidal signs.^9^

In this study, we set out to investigate the P436S mutation in *PSEN1*. Despite two individuals with memory impairment having been previously described with this mutation,^19^ the pathogenicity of P436S remains unclassified (alzforum.org). Here, using an expanded kindred, clinical findings, post‐mortem brain tissue, and iPSC models, we tested the hypothesis that *PSEN1* P436S is associated with fAD, and further characterize its clinical manifestations.

**RESEARCH IN CONTEXT**

1. **Systematic review**: Relatively little is known regarding the *PSEN1* P436S mutation in familial Alzheimer's disease (fAD). The authors performed a literature search on the P436S mutation and mutations in proximity. The relevance of mutations in the PALP motif of *PSEN1*, in which this mutation lies, is reviewed.
2. **Interpretation**: Findings confirm the pathogenicity of the *PSEN1* P436S mutation in fAD. Atypical clinical manifestations and elevated amyloid beta 43 (Aβ43) production are described. The early age at clinical onset supports the importance of the PALP motif in proper PSEN1 function.
3. **Future directions**: This study reinforces the need for further clinical and molecular studies into the heterogeneity of fAD. Investigations may compare the biochemical effects of P436S with P436Q, fAD mutations with ages at onset that differ by 18 years. Future work is required to confirm and investigate the finding that generation of all Aβ species is increased by the P436S mutation.

## 2 METHODS

### 2.1 Subjects

**FIGURE 1:**
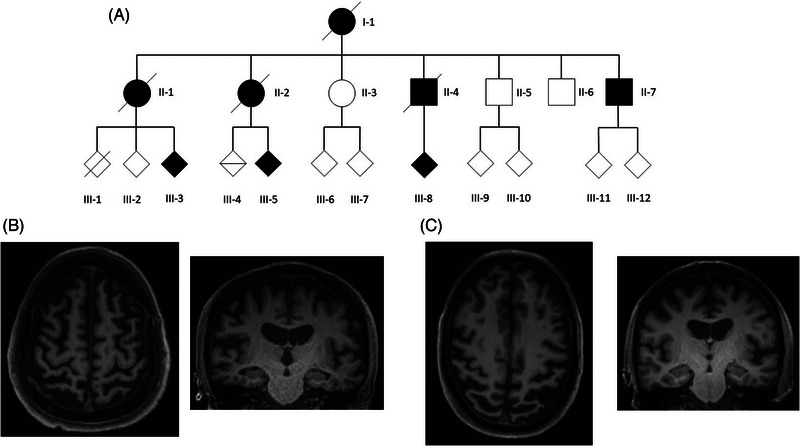
*PSEN1* P436S expanded family kindred and MRI findings support autosomal dominant fAD. (A) Updated kindred of family. II‐4 is the individual reported previously by Palmer et al.^19^ (B) Example MRI images from individual III‐5. T1 axial and coronal images are shown at age 48, demonstrating generalized atrophy and low‐volume hippocampi. (C) Example MRI images from individual III‐8. T1 axial and coronal images are shown at age 51, demonstrating posterior cerebral atrophy, widening of the left Sylvian fissure, and relatively preserved hippocampal volume.

We provide an updated three‐generation pedigree of a family with *PSEN1* P436S associated fAD. Clinical information was obtained from research and clinical contact. Post‐mortem results were available for one individual (individual II‐4, Figure 1).

Fibroblast biopsies were taken with research ethics approval (11/LO/0753) and with informed consent. Biopsies were donated by individuals III‐5 and III‐8, both female. III‐5 was 44 years at symptom onset and 48 years at the time of biopsy. III‐8 was 48 years at symptom onset and 52 years at time of biopsy.

### 2.2 Cell culture

Fibroblasts were grown in Dulbecco's modified eagle media (DMEM) with 10% fetal bovine serum and passaged using 0.05% trypsin. Fibroblasts were reprogrammed using Epi5 plasmids, obtained from Thermo Fisher. A Lonza P2 nucleofector was used to electroporate the plasmids into 750,000 fibroblasts. Media was changed from fibroblast media to Essential 8 iPSC media after passaging the fibroblasts onto Geltrex substrate on day 7. Colonies emerged after 4–5 weeks and were picked, expanded, and banked for characterization. iPSCs were cultured routinely in Essential 8 media on Geltrex substrate and passaged using 0.5 mM EDTA at a ratio of around 1:6 every 5 days.

Details of the control iPSC lines and fAD patient‐derived lines are shown in Table 1.

**TABLE 1:** iPSC lines used in this study.

Line	Details and reference	Female/male	Age at biopsy	*APOE* status
Control 1	KOLF2.1J ^20^	Male	55–59	3/3
Control 2	RBi01 (Sigma) ^13^	Male	45–49	3/3
*PSEN1* int4del	EBiSC ^13^	Female	47	3/3
*PSEN1* Y115H	*PSEN1* Y115H ^13^	Male	39	3/3
*PSEN1* R278I	*PSEN1* R278I cA ^13^	Male	60	2/4
*PSEN1* E280G	*PSEN1* E280G.A ^17^	Male	45	3/3
*PSEN1* P436S #1	III‐5–this study	Female	52	2/3
*PSEN1* P436S #2	III‐8–this study	Female	48	3/3

Abbreviation: iPSC, induced pluripotent stem cell.

Neuronal differentiation was performed as reported previously.^13^, ^21^, ^22^ Briefly, iPSCs were grown to confluence and then switched to N2B27 media with 10 µM SB431542 and 1 µM dorsomorphin. After 10 days of neural induction, neural precursors were passaged onto laminin in N2B27 media. A final passage of progenitors was performed on day 35 onto laminin and poly‐ornithine coated wells. The cells were allowed to terminally differentiate and mature until day 100, which was taken as the final time point.

All reagents were Thermo Fisher unless stated otherwise.

### 2.3 Sanger sequencing

Sanger sequencing was performed with SourceBio on polymerase chain reaction (PCR) amplicons (GoTaq, Promega) of genomic DNA using the following primers: forward primer GTCTTTCCCATCTTCTCCAC and reverse primer GGGATTCTAACCGCAAATAT.

### 2.4 qPCR‐based karyotyping

Quantitative PCR (qPCR)–based karyotyping was performed using the Stem Cell Technology human pluripotent stem cell genetic analysis kit. This tests for the eight most common chromosomal amplifications/deletions in stem cell cultures.

### 2.5 qPCR

RNA was extracted from neurons using Trizol and RNA precipitation. A total of 2 µg of RNA was reverse transcribed to complementary DNA using Superscript IV following manufacturer's instructions. qPCR was performed using POWER Sybr green using the following primers all with annealing temperatures at 60°C: housekeeping gene *RPL18A* forward CCCACAACATGTACCGGGAA and *RPL18A* reverse TCTTGGAGTCGTGGAACTGC; *TBR1* forward AGCAGCAAGATCAAAAGTGAGC and *TBR1* reverse ATCCACAGACCCCCTCACTAG; *CTIP2* forward CTCCGAGCTCAGGAAAGTGTC and *CTIP2* reverse TCATCTTTACCTGCAATGTTCTCC; *TUBB3* forward CATGGACAGTGTCCGCTCAG and *TUBB3* reverse CAGGCAGTCGCAGTTTTCAC; *APP* forward GGTACCCACTGATGGTAAT and *APP* reverse GGTAGACTTCTTGGCAATAC; *PSEN1* forward TATCAAGTACCTCCCTGAAT and *PSEN1* reverse ACCATTGTTGAGGAGTAAAT; and *PSEN2* forward GACTCCTATGACAGTTTTGG and *PSEN2* reverse GCACACTGTAGAAGATGAAGT.

### 2.6 Immunocytochemistry

Cells were plated on glass coverslips and fixed using 4% paraformaldehyde for 15 min at room temperature. Cells were then permeabilized in phosphate‐buffered saline (PBS) with 0.3% Triton‐X‐100 (hereafter PBSTx) via three washes. Cells were then blocked in 3% bovine serum albumin (BSA) in PBSTx for 30 min at room temperature. Primary antibodies were added in blocking solution overnight at 4°C. Cells were then washed three times in PBSTx, and secondary antibodies (AlexaFluor) were added in blocking solution for 1 h at room temperature. Cells were washed a final three times in PBSTx and counterstained using DAPI. Finally, cells were mounted, using DAKO fluorescence mounting media and imaged on a Zeiss LSM microscope with no post hoc adjustments to the images.

Primary antibodies were: SSEA4 Biolegend 330401 RRID:AB_1089209; NANOG Cell Signalling Technology 4903 RRID:AB_10559205; TBR1 Abcam ab31940 RRID:AB_2200219; and TUJ1 Biolegend 801201 RRID:AB_2313773.

### 2.7 Immunohistochemistry

Post‐mortem brain tissue from individual II‐4 assessed in this study was obtained through the brain donation program of the London Neurodegenerative Diseases Brain Bank. The protocols used for brain donation and ethical approval for this study were approved by a London Research Ethics Committee and tissue is stored for research under a license from the Human Tissue Authority. The standard diagnostic criteria for the neuropathological diagnosis of AD were used.^23^, ^24^, ^25^ Slides with 8 µm paraffin‐embedded tissue sections from the temporal cortex were incubated at 60°C overnight. Sections were deparaffinized in xylene and rehydrated in decreasing grades of alcohol. Slides were incubated in methanol/hydrogen peroxide (0.3%) solution for 10 min to block endogenous peroxidase activity. Slides were then incubated in 98% formic acid for 15 min. For heat‐induced antigen retrieval, slides were then transferred to a boiling solution of 0.1 M citrate buffer (pH 6.0) and pressure cooked at maximum pressure for 10 min. Subsequently sections were incubated in 1% BSA for 30 min at room temperature to block non‐specific binding. Sections were incubated with primary antibody for 2 h at room temperature. The antibodies used for immunohistochemistry in this study were mouse‐derived monoclonal anti‐β‐amyloid, 1‐43 (BioLegend 805607, 1:500), anti‐β‐amyloid, clone 6F/3D (DAKO, M0872, 1:200) and AT8 anti‐phosphorylated tau (Thermo Scientific MN1020, 1:600). After three 5 min washes in tris‐buffered saline with 0.1% Tween‐20 (TBS‐Tw), slides were incubated for 1 h in biotinylated goat anti‐mouse IgG secondary antibody (Vector Laboratories BA 9200, 1:200). Slides were washed as before and then incubated in pre‐conjugated Strept(avidin)–Biotin Complex (ABC; DAKO) for signal amplification. The slides were then washed for a final time before being submerged in 3,3ʹ‐diaminobenzidine (DAB) chromogen and then counterstained in Mayer's haematoxylin (BDH). Finally, slides were dehydrated in increasing grades of alcohol (70, 90, and 100% IMS), cleared in xylene, and mounted. Tissue sections were digitally scanned using an Olympus VS120 slide scanner at 20 × magnification.

### 2.8 Western blotting

Cells were lysed in RIPA buffer containing protease and phosphatase inhibitors (Roche). Lysates were centrifuged to remove insoluble debris and protein content was quantified using BCA assay (BioRad). Samples were standardized for protein content and denatured using LDS buffer and dithiothreitol with boiling at 95°C for 5 min. Proteins were separated using 4%–12% precast polyacrylamide gels and transferred onto nitrocellulose membranes. Membranes were blocked in PBS with 0.1% Tween‐20 (PBSTw) with 3% BSA. Primary antibodies were added overnight at 4°C in blocking solution. Membranes were then washed three times and secondary antibodies (Licor) were added for 1 h in blocking solution. A final three washes were performed prior to imaging on a Licor Odyssey scanner.

Primary antibodies were: PSEN1 NTF Millipore MAB1563 RRID: B_11215630; sAPPβ IBL JP18957 RRID:AB_1630824; sAPP total (22c11) Millipore MAB348 RRID:AB_94882; APP ctf Thermo A8717 RRID:AB_258409; PSEN2 Cell Signalling Technology 9979 RRID:AB_10829910; and Actin Sigma A1978 RRID:AB_476692.

### 2.9 Aβ ELISAs

Media was harvested after 48 h conditioning on iPSC‐derived neurons. Conditioned media was centrifuged at 2000 × *g* for 5 min to remove cellular debris. Aβ38, Aβ40, and Aβ42 were quantified simultaneously via Meso Scale Discovery V‐PLEX Aβ peptide panel (6E10). Samples were diluted two‐fold and measurements were made against standard curves on an MSD Sector 6000. Aβ43 was quantified using IBL Amyloid Beta (1‐43) (FL) enzyme‐linked immunosorbent assay (ELISA). Media was run undiluted against a standard curve following manufacturer's protocols. Measurements were made on a TECAN SPARK 10 M plate reader.

### 2.10 Statistical analyses

Statistical comparisons were performed using Microsoft Excel and GraphPad Prism. Normality was tested using Shapiro–Wilk test and data were compared to pooled controls using one‐way analysis of variance (ANOVA) with Dunnett's multiple comparison test. Data are shown as * = *p* < .05 , ** = *p* < .01, *** = *p* < .001, and **** = .*p* < .0001

## 3 RESULTS

### 3.1 Expanded kindred and case descriptions

We provide an updated three‐generation pedigree of a family with *PSEN1* P436S (Figure 1A). Clinical information was obtained from research and clinical contact (Table 2).

**TABLE 2:** Case descriptions of the expanded *PSEN1* P436S kindred.

Individual	Age at onset	Age at death	Clinical observations
I‐1	Unknown	49	Diagnosed with “pre‐senile dementia.”
II‐1	45	50	Initial memory led to decline and apraxia. On examination at age 47, pathologic hyperreflexia in the lower limbs and diffuse cortical atrophy noted on CT scan.
II‐2	47	68	Initial memory impairment. MMSE at age 53 was 22/30, with impaired recall, calculation, sentence and figure copying. There was late development of hallucinations and seizures.
II‐4	44	52	Initial memory impairment. MMSE age 47 was 7/30, with severe verbal, visual and general memory, and attention impairment on psychometry. CT of the brain demonstrated moderate generalized atrophy. Post‐mortem confirmation of AD neuropathologic change (see Figure 3). Confirmed carrier of *PSEN1* P436S. *APOE* status ε3/ε3.
II‐7	47	N/A	Limited information.
III‐3	46	N/A	Initial impairment in memory with word‐finding difficulties and apraxia.
III‐5	44	N/A	Initial memory impairment. At age 46 they developed apraxia and dyscalculia. On examination age 48 there was apraxia, myoclonus, increased tone in the lower limbs with hyper‐reflexia and downgoing plantar responses, and no parkinsonism or cerebellar signs. MRI at age 48 demonstrated generalized atrophy including the hippocampi (Figure 1B). At age 49 they developed word‐finding difficulty and a stutter. Confirmed carrier of *PSEN1* P436S. *APOE* status ε2/ε3.
III‐8	48	N/A	Initial executive dysfunction with slowed processing and planning, and then developed topographic memory dysfunction, apraxia, and language dysfunction with word‐finding difficulties. On examination at age 51 there was apraxia, spasticity, and hyper‐reflexia in the lower limbs with downgoing plantar responses and subtle myoclonus. MMSE was 14/30. MRI at age 51 demonstrated posterior atrophy (Figure 1C). They developed agitation at age 53 treated with risperidone, and on examination at age 54 there was parkinsonism with hypomimia, right‐sided rest tremor, rigidity, and bradykinesia. They developed seizures at age 55. Confirmed carrier of *PSEN1* P436S. *APOE* status ε3/ε3.

Abbreviations: MMSE, mini mental state examination; CT, computed tomography; MRI, magnetic resonance imaging.

The kindred are consistent with an autosomal dominant pattern of inheritance (Figure 1A). The average age onset is 46 years (44–50), most commonly with a memory led decline, and with apraxia as a common associated feature. Leading executive dysfunction was observed in one individual. Lower limb pyramidal signs or spastic paraparesis as a late manifestation was observed frequently, in three of the five affected individuals with documented neurological examinations. MRI was available for two individuals (Figure 1B,C) and demonstrated generalized cerebral atrophy including the hippocampi for individual III‐5, and marked posterior atrophy with relatively preserved hippocampal volume for individual III‐8. Neither had white matter hyperintensities on fluid‐attenuated inversion recovery (FLAIR) or cerebral microbleeds on susceptibility‐weighted imaging (SWI). Together, these findings support the hypothesis that *PSEN1* P436S mutations cause fAD, with observed atypical features in some individuals including late spastic paraparesis. Additionally, one individual showed non‐episodic memory lead cognitive decline in combination with significant posterior atrophy on imaging.

### 3.2 iPSC models demonstrate elevated Aβ43:40 ratios in P436S mutant neurons

**FIGURE 2:**
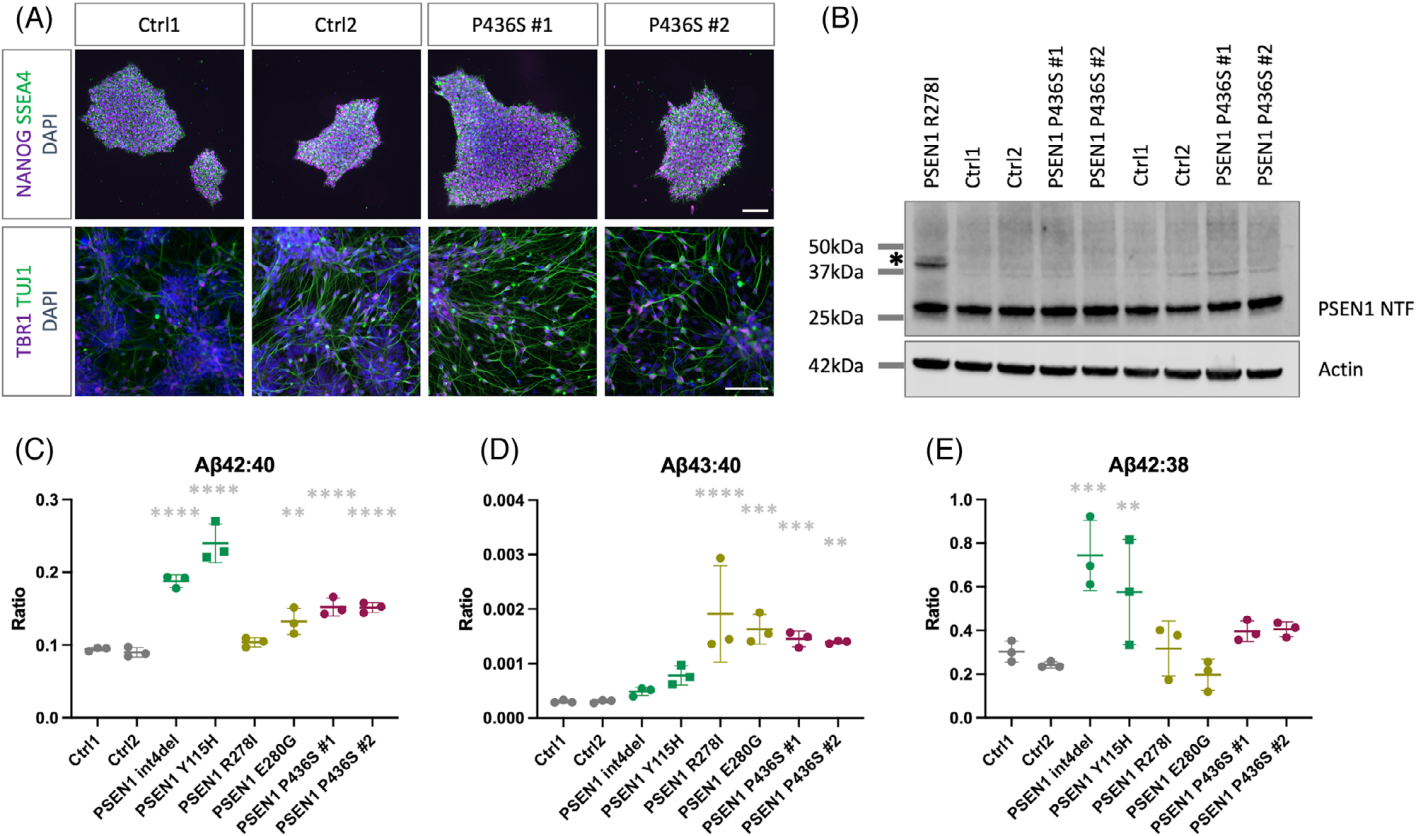
*PSEN1* P436S patient‐derived iPSC neuron models display fAD‐associated Aβ profiles. (A) Immunocytochemical analysis of iPSCs (top) confirming expression of pluripotency markers NANOG and SSEA4. Immunocytochemical analysis of iPSC‐derived neurons at day 50 of differentiation, bottom, displaying neuronal morphology, expression of deep layer cortical marker TBR1, and neuronal‐specific tubulin TUJ1. (B) Western blot analysis of PSEN1 maturation in neuronal lysates collected after 100 days of differentiation. Immature PSEN1 at 48 kDa (*) is cleaved to a mature N‐terminal fragment (NTF) at around 28 kDa. *PSEN1* R278I is displayed as a mutation that causes maturation defects. Actin is used as a loading control. Two (of three) independent inductions are shown (all three replicates are shown in Figure S2A). (C–E) Aβ peptide analysis by electrochemiluminescence to depict the disease‐associated Aβ42:40 ratio, the Aβ43:40 ratio, and the processivity ratio Aβ42:38. Int4del and Y115H (green) display typical *PSEN1* mutation‐associated ratio changes; R278I and E280G (yellow) are shown as high Aβ43 producing mutations, each serving as comparators to control (gray) and P436S (red) lines. P436S lines display significantly increased Aβ42:40 and Aβ43:40 ratios. Data represent three independent inductions for each line. Significance represents one‐way ANOVA and Dunnett's multiple comparisons test for each patient‐derived line versus pooled controls, ** = *p* < .01, *** = *p* < .001, **** = *p* < .0001. fAD, Familial Alzheimer's disease; iPSC, induced pluripotent stem cells.

In order to generate a human neuronal model of the P436S mutation, fibroblasts from two donors were reprogrammed to iPSCs (III‐5 and III‐8, see Methods). Newly generated iPSCs were confirmed to have iPSC morphology and expression of pluripotency‐associated factors NANOG and SSEA4 by immunocytochemistry (Figure 2A). The presence of the P436S mutation was confirmed by Sanger sequencing and the iPSC lines were shown to have a stable karyotype (Figure S1A,B). Together with two control iPSC lines, cells were differentiated to cortical, glutamatergic neurons. Patient‐derived lines generated iPSC‐derived neurons with a similar efficiency to control lines, suggesting that the mutation did not affect neuronal differentiation (Figure 2A and Figure S1C–E).

PSEN1 undergoes autoproteolysis from an immature full‐length peptide of ∼48 kDa to two mature peptides of ∼28 and 18 kDa. Some mutations associated with atypical presentations of fAD have shown deficits in PSEN1 protein maturation.^13^, ^17^ To investigate the maturation of P436S‐variant PSEN1, western blotting of neuronal lysates was performed. Uncleaved, immature PSEN1 is observed at ∼48 kDa, whereas mature, cleaved PSEN1 is observed at ∼28 kDa. In contrast to PSEN1 R278I, results suggested appropriate processing of the PSEN1 P436S peptide to mature peptide fragments (Figure 2B).

Analysis of Aβ species released into the culture media by the iPSC‐derived neurons demonstrated that both lines with the P436S mutation displayed a raised Aβ42:40 ratio and a large increase in the Aβ43:40 ratio (Figure 2C,D). The processivity‐associated Aβ42:38 ratio was similar in P436S neurons compared with controls (Figure 2E). When normalized for cell numbers, all Aβ species measured showed an increased concentration in the cell culture media compared to other mutations (Figure S1F–I). This contrasts with previously studied *PSEN1* mutations, which show decreased generation of shorter Aβ peptides such as Aβ38, attributable to reduced processivity (Figure S1F,I). *APP* expression, *PSEN1* expression, and amyloidogenic versus non‐amyloidogenic processing were not altered and, therefore, could not explain this finding (Figure S2). We do, however, note a degree of variability between control lines.

Together, these data support the finding that *PSEN1* P436S leads to impaired Aβ processing and high Aβ43 production, similar to some other fAD mutations with atypical clinical presentations.

### 3.3 Pathological diagnosis and confirmation of Aβ43 pathology in a P436S carrier

**FIGURE 3:**
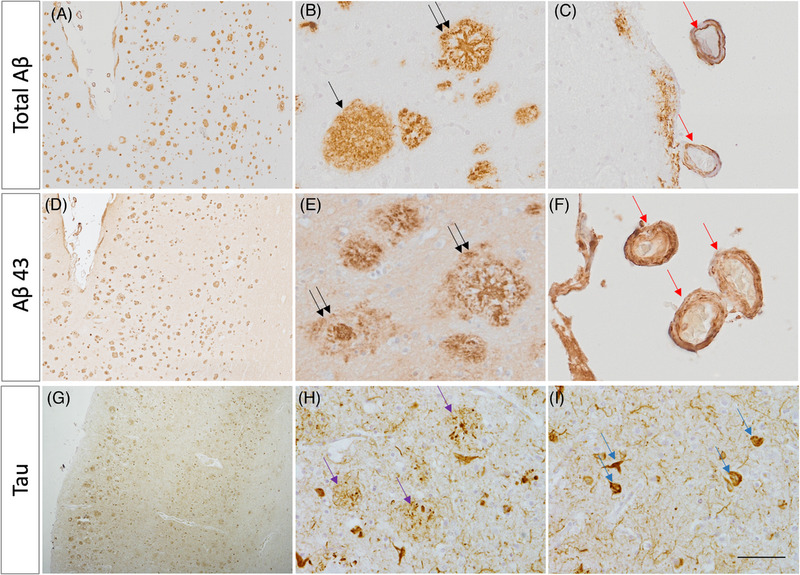
Immunohistochemical staining of post‐mortem tissue displays Aβ43 deposition. (A–C) Total Aβ staining shows plaque pathology (A, B) and amyloid angiopathy (C). Cotton wool plaques (black single arrow), senile/cored plaques (black double arrow), and leptomeningeal cerebral amyloid angiopathy (CAA; red arrows) are depicted. (D–E) Aβ43‐specific staining demonstrates substantial Aβ43 deposition in both parenchymal plaques and in vessels as cerebral amyloid angiopathy. Senile cored plaques (black double arrows) and leptomeningeal CAA (red arrows) are depicted. (G–I) AT8 immunostaining for phosphorylated tau, demonstrating neuritic plaques (purple arrows) and neurofibrillary tangles (blue arrows). Scale bar represents 500 µm in A, D, and G; and 50 µm in B, C, E, F, H, and I.

Microscopic investigation of individual II‐4 confirmed the presence of severe AD‐type pathology, using the National Institute on Aging/Alzheimer's Association (NIA/AA) criteria. Microscopic investigations demonstrated frequent and widespread deposition of Aβ in parenchymal plaques (corresponding to Thal phase 5); the neuritic plaque pathology was of CERAD ‘frequent’ degree and also present in leptomeningeal and parenchymal blood vessels, indicative of severe cerebral amyloid angiopathy (Figure 3). Hemorrhagic events were not noted. The tau pathology was very severe throughout including the striate and peristriate cortices. The neurofibrillary tangle pathology, therefore, corresponded to Braak stage VI. In summary, this case had “High” AD neuropathological change with an NIA/AA score of A3B3C3. In addition, there was also evidence of amygdala predominant Lewy body disease. There was no evidence of TDP43 pathology.

To confirm the finding that *PSEN1* P436S mutant neurons produce high levels of Aβ43, we also performed immunohistochemical analysis for total Aβ and Aβ43 on post‐mortem brain tissue from the temporal cortex (Figure 3). Analysis suggests that Aβ43 represents a major component of the amyloid plaques in P436S‐associated tissue. Aβ43 staining highlighted dense cores within plaques as well as extensive cerebral amyloid angiopathy. There was also evidence for cotton wool plaque pathology (round lesions that lack central dense cores that have been linked with atypical *PSEN1* mutations), although cotton wool plaques and dense core plaques were present with similar abundance. These data confirm that Aβ43 pathology is associated with the *PSEN1* P436S mutation.

## 4 DISCUSSION

Making use of an expanded family history, MRI brain scans, iPSC models, and post‐mortem tissue, we confirm that the P436S mutation in *PSEN1* is causative of fAD and describe its atypical clinical features.

This kindred highlights the heterogeneity that can be observed in fAD despite a common causative mutation, and the potential atypical cognitive presentations and motor syndromes, particularly with certain *PSEN1* mutations.^9^ Although the age at symptom onset was relatively consistent (range 44–48), the disease duration (range 5–21) and age at death (range 49–68) varied widely. This is in keeping with previous work in fAD demonstrating that survival time is influenced by mutation to a lesser extent than age at onset.^26^ In most cases, the cognitive syndrome was memory led (4/5), but one individual (III‐8) presented with an atypical cognitive manifestation of initial executive dysfunction (a clinical phenotype that can be seen as an atypical manifestation of fAD and sporadic early onset AD),^9^, ^27^, ^28^ and demonstrated significant posterior cerebral atrophy on imaging with relative preservation of the hippocampi (radiologically similar to that which is seen in posterior cortical atrophy [PCA], another atypical clinical phenotype of early onset AD that is held to be sporadic,^29^ although notably, this individual did not have the clinical syndrome of PCA). Distinct from sporadic AD and fAD caused by *APP* mutations, in this kindred, spastic paraparesis was observed as a late manifestation in a majority of cases with documented neurological examinations (3/5), and this study further reinforces the need to consider *PSEN1* mutations in individuals presenting with cognitive decline and spastic paraparesis.^30^ Late parkinsonism was seen in one individual, although this could be confounded by neuroleptic exposure. Hemorrhage was not noted on the neuropathological report, and although CAA is commonly observed on neuropathological examination of individuals with fAD, cerebral microbleeds are seen on MRI in a far smaller proportion of patients and hemorrhagic events are rare.^31^

The link between Aβ43 and atypical motor manifestations warrants attention in future clinical studies. Elevated Aβ43 has been reported in other fAD mutations that are associated with spastic paraparesis, including E280G and R278I,^13^, ^17^ and with parkinsonism, including L435F.^32^, ^33^ Our study also supports the correlation between *PSEN1* mutations associated with spastic paraparesis and cotton wool plaque pathology, which in turn has been shown to correlate with cerebral amyloid angiopathy in some, but not all, cases.^33^, ^34^ The pathophysiology of spastic paraparesis in *PSEN1*‐associated fAD is not well understood and may relate to altered processing of γ‐secretase substrates other than APP.^30^ The apparent association between increased Aβ43 and spastic paraparesis may potentially represent a signature of altered γ‐secretase function as it relates to these other substrates. Relevant substrates may become clear as knowledge of γ‐secretase substrates continues to expand.^35^ The two individuals from whom iPSCs were derived exhibited differing clinical and radiologic manifestations despite similar Aβ peptide profiles. This reinforces the need to consider factors other than Aβ processing in the clinical heterogeneity of fAD in general.

The PSEN1 P436S peptide variant shows appropriate autoproteolysis and maturation, shown via the absence of an immature PSEN1 band by western blotting. This replicates findings in mouse embryonic fibroblasts and cell models, whereby P436S shows largely normal processing, which is in contrast to the P436Q mutation.^33^, ^36^ The current work disputes a previous correlation between incomplete PSEN1 peptide maturation and Aβ43 production for the R278I and E280G mutations.^13^, ^17^

The P436 residue of PSEN1 lies within the PALP motif (residues P433, A434, L435, and P436; reviewed by Bagaria, Bagyinszky, and An, 2022^37^). Stereologically, the PALP motif lies close to the catalytic pore of PSEN1 and is critical for proper enzymatic function.^38^, ^39^ The importance of this region may explain the early age at onset of mutations associated with this motif. P433S leads to age at onset of 34 years,^36^ A434C and A434T have ages of onset between 29 and 35 years, respectively,^40^, ^41^ L435F has an age of onset of 47,^42^ and P436Q has an age at onset of 28.^43^ Therefore, we theorize that the P436S mutation may have a less severe impact on PSEN1 function compared to P436Q (which shows defective PSEN1 autoproteolysis, a very early age at onset, and is also associated with spastic paraparesis and posterior cerebral clinical features ^33^, ^43^) yet still cause fAD with a relatively early age at onset due to the importance of the PALP motif. Proline residues provide structural rigidity and are often crucial to peptide turns. This potentially highlights (1) the importance of the PALP motif close to the ninth transmembrane domain of PSEN1, and (2) a more detrimental effect of the larger glutamine residue in P436Q compared to the smaller serine substitution in P436S. Finally, we were surprised to see increased levels of all Aβ species measured in P436S conditioned media. This finding supports data from cell models,^36^ which show increased Aβ40, Aβ42, and Aβ43 production, but contradicts data in *PSEN1/2* double knockout fibroblasts.^33^ The presence/absence of the healthy *PSEN1* allele represents a point of difference between the studies. Further experiments are required to test the basis of this finding.

One of the limitations of this study is the rarity of fAD and of the *PSEN1* P436S mutation, affecting the number of patient‐derived iPSC lines and post‐mortem tissue available. The use of isogenic control lines would further confirm the findings. Further experiments are required to confirm and determine the mechanism of raised total Aβ species in the media of P436S neurons; these may include investigating intracellular Aβ levels or measuring Aβ flux using methods such as stable isotope labeling.^44^ Finally, it has been suggested that fAD mutations associated with high Aβ43 cause processing pathway switching, and that Aβ43 is generated from Aβ48 rather than Aβ46.^45^ Further work may assess this phenomenon in *PSEN1* P436S patient‐derived neurons.

In summary, this study confirms the pathogenicity of the *PSEN1* P436S mutation, validating a causative effect for fAD. The data further underscore the relevance of Aβ43 in the pathogenesis of fAD and the association between Aβ43 and atypical motor manifestations, and support a critical importance of the PALP domain in PSEN1 enzymatic function. Together these findings further develop our molecular understanding of fAD and highlight the importance of considering clinical heterogeneity when designing clinical studies.

## CONFLICT OF INTEREST STATEMENT

The authors have no conflicts to declare. Author disclosures are available in the supporting information

## Supporting information

Supporting information

Supporting information

## References

[1] Goate A , Chartier‐Harlin MC , Mullan M , et al. Segregation of a missense mutation in the amyloid precursor protein gene with familial Alzheimer's disease. Nature. 1991;349(6311):704‐706. doi:10.1038/349704a0 1671712

[2] Levy‐Lahad E , Wasco W , Poorkaj P , et al. Candidate gene for the chromosome 1 familial Alzheimer's disease locus. Science. 1995;269(5226):973‐977. doi:10.1126/science.7638622 7638622

[3] Sherrington R , Rogaev EI , Liang Y , et al. Cloning of a gene bearing missense mutations in early‐onset familial Alzheimer's disease. Nature. 1995;375(6534):754‐760. doi:10.1038/375754a0 7596406

[4] Selkoe DJ , Hardy J . The amyloid hypothesis of Alzheimer's disease at 25 years. EMBO Mol Med. 2016;8(6):595‐608. doi:10.15252/emmm.201606210 27025652 PMC4888851

[5] Chávez‐Gutiérrez L , Bammens L , Benilova I , et al. The mechanism of γ‐Secretase dysfunction in familial Alzheimer disease. EMBO J. 2012;31(10):2261‐2274. doi:10.1038/emboj.2012.79 22505025 PMC3364747

[6] Matsumura N , Takami M , Okochi M , et al. γ‐Secretase associated with lipid rafts: multiple interactive pathways in the stepwise processing of β‐carboxyl‐terminal fragment. J Biol Chem. 2014;289(8):5109‐5121. doi:10.1074/jbc.M113.510131 24375443 PMC3931069

[7] Takami M , Nagashima Y , Sano Y , et al. gamma‐Secretase: successive tripeptide and tetrapeptide release from the transmembrane domain of beta‐carboxyl terminal fragment. J Neurosci. 2009;29(41):13042‐13052. doi:10.1523/JNEUROSCI.2362-09.2009 19828817 PMC6665297

[8] Szaruga M , Munteanu B , Lismont S , et al. Alzheimer's‐causing mutations shift Aβ length by destabilizing γ‐Secretase‐Aβn interactions. Cell. 2017;170(3):443‐456. e14. doi:10.1016/j.cell.2017.07.004 28753424

[9] Ryan NS , Nicholas JM , Weston PSJ , et al. Clinical phenotype and genetic associations in autosomal dominant familial Alzheimer's disease: a case series. Lancet Neurol. 2016;15(13):1326‐1335. doi:10.1016/S1474-4422(16)30193-4 27777022

[10] Ryman DC , Acosta‐Baena N , Aisen PS , et al. Symptom onset in autosomal dominant Alzheimer disease: a systematic review and meta‐analysis. Neurology. 2014;83(3):253‐260. doi:10.1212/WNL.0000000000000596 24928124 PMC4117367

[11] Verkkoniemi A , Somer M , Rinne JO , et al. Variant Alzheimer's disease with spastic paraparesis: clinical characterization. Neurology. 2000;54(5):1103‐1109. doi:10.1212/wnl.54.5.1103 10720282

[12] Arber C , Lovejoy C , Wray S . Stem cell models of Alzheimer's disease: progress and challenges. Alzheimers Res Ther. 2017;9(1):42. doi:10.1186/s13195-017-0268-4 28610595 PMC5470327

[13] Arber C , Toombs J , Lovejoy C , et al. Familial Alzheimer's disease patient‐derived neurons reveal distinct mutation‐specific effects on amyloid beta. Mol Psychiatry. 2020;25(11):2919‐2931. doi:10.1038/s41380-019-0410-8 30980041 PMC7577860

[14] O'Connor A , Pannee J , Poole T , et al. Plasma amyloid‐β ratios in autosomal dominant Alzheimer's disease: the influence of genotype. Brain. 2021;144(10):2964‐2970. doi:10.1093/brain/awab166 33892504 PMC8634092

[15] Saito T , Suemoto T , Brouwers N , et al. Potent amyloidogenicity and pathogenicity of Aβ43. Nat Neurosci. 2011;14(8):1023‐1032. doi:10.1038/nn.2858 21725313

[16] Oakley DH , Chung M , Klickstein N , Commins C , Hyman BT , Frosch MP . The Alzheimer disease‐causing presenilin‐1 L435F mutation causes increased production of soluble Aβ43 species in patient‐derived iPSC‐neurons, closely mimicking matched patient brain tissue. J Neuropathol Exp Neurol. 2020;79(6):592‐604. doi:10.1093/jnen/nlaa025 32388561 PMC7241938

[17] Willumsen N , Arber C , Lovejoy C , et al. The PSEN1 E280G mutation leads to increased amyloid‐β43 production in induced pluripotent stem cell neurons and deposition in brain tissue. Brain Commun. 2022;5(1):fcac321. doi:10.1093/braincomms/fcac321 36687397 PMC9847549

[18] Hurley EM , Mozolewski P , Dobrowolski R , Hsieh J . Familial Alzheimer's disease‐associated PSEN1 mutations affect neurodevelopment through increased Notch signaling. Stem Cell Reports. 2023;18(7):1516‐1533. doi:10.1016/j.stemcr.2023.05.018 37352850 PMC10362499

[19] Palmer MS , Beck JA , Campbell TA , et al. Pathogenic presenilin 1 mutations (P436S & I143F) in early‐onset Alzheimer's disease in the UK. Hum Mutat. 1999;13(3):256‐256. doi:10.1002/(SICI)1098-1004(1999)13:3<;256::AID-HUMU11>;3.0.CO;2-P 10090481

[20] Pantazis CB , Yang A , Lara E , et al. A reference human induced pluripotent stem cell line for large‐scale collaborative studies. Cell Stem Cell. 2022;29(12):1685‐1702. e22 doi:10.1016/j.stem.2022.11.004 36459969 PMC9782786

[21] Arber C , Villegas‐Llerena C , Toombs J , et al. Amyloid precursor protein processing in human neurons with an allelic series of the PSEN1 intron 4 deletion mutation and total presenilin‐1 knockout. Brain Commun. 2019;1(1):fcz024. doi:10.1093/braincomms/fcz024 32395715 PMC7212081

[22] Arber C , Lovejoy C , Harris L , et al. Familial Alzheimer's disease mutations in PSEN1 lead to premature human stem cell neurogenesis. Cell Rep. 2021;34(2):108615. doi:10.1016/j.celrep.2020.108615 33440141 PMC7809623

[23] Braak H , Braak E , Neuropathological stageing of Alzheimer‐related changes. Acta Neuropathol 1991;82(4):239‐259. doi:10.1007/BF00308809 1759558

[24] Hyman BT , Phelps CH , Beach TG , et al. National Institute on Aging‐Alzheimer's Association guidelines for the neuropathologic assessment of Alzheimer's disease. Alzheimers Dement. 2012;8(1):1‐13. doi:10.1016/j.jalz.2011.10.007 22265587 PMC3266529

[25] Thal DR , Rüb U , Orantes M , Braak H . Phases of A beta‐deposition in the human brain and its relevance for the development of AD. Neurology. 2002;58(12):1791‐1800. doi:10.1212/wnl.58.12.1791 12084879

[26] Pavisic IM , Nicholas JM , O'Connor A , et al. Disease duration in autosomal dominant familial Alzheimer disease: a survival analysis. Neurol Genet. 2020;6(5):e507. doi:10.1212/NXG.0000000000000507 33225064 PMC7673285

[27] Townley RA , Graff‐Radford J , Mantyh WG , et al. Progressive dysexecutive syndrome due to Alzheimer's disease: a description of 55 cases and comparison to other phenotypes. Brain Commun. 2020;2(1):fcaa068. doi:10.1093/braincomms/fcaa068 32671341 PMC7325839

[28] Ossenkoppele R , Pijnenburg YAL , Perry DC , et al. The behavioural/dysexecutive variant of Alzheimer's disease: clinical, neuroimaging and pathological features. Brain. 2015;138(Pt. 9):2732‐2749. doi:10.1093/brain/awv191 26141491 PMC4623840

[29] Schott JM , Crutch SJ . Posterior Cortical Atrophy. Continuum. 2019;25(1):52‐75. doi:10.1212/CON.0000000000000696 30707187 PMC6548537

[30] Chelban V , Breza M , Szaruga M , et al. Spastic paraplegia preceding PSEN1‐related familial Alzheimer's disease. Alzheimers Dement. 2021;13(1):e12186. doi:10.1002/dad2.12186 PMC8088589 33969176

[31] Banerjee G , Collinge J , Fox NC , et al. Clinical considerations in early‐onset cerebral amyloid angiopathy. Brain. 2023;146(10):3991‐4014. doi:10.1093/brain/awad193 37280119 PMC10545523

[32] Kretner B , Trambauer J , Fukumori A , et al. Generation and deposition of Aβ43 by the virtually inactive presenilin‐1 L435F mutant contradicts the presenilin loss‐of‐function hypothesis of Alzheimer's disease. EMBO Mol Med. 2016;8(5):458‐465. doi:10.15252/emmm.201505952 26988102 PMC5119496

[33] Heilig EA , Xia W , Shen J , Kelleher RJ . A presenilin‐1 mutation identified in familial Alzheimer disease with cotton wool plaques causes a nearly complete loss of γ‐secretase activity. J Biol Chem. 2010;285(29):22350‐22359. doi:10.1074/jbc.M110.116962 20460383 PMC2903357

[34] Willumsen N , Poole T , Nicholas JM , Fox NC , Ryan NS , Lashley T . Variability in the type and layer distribution of cortical Aβ pathology in familial Alzheimer's disease. Brain Pathol. 2022;32(3):e13009. doi:10.1111/bpa.13009 34319632 PMC9048809

[35] Hou P , Zielonka M , Serneels L , et al. The γ‐secretase substrate proteome and its role in cell signaling regulation. Mol Cell. 2023;83(22):4106‐4122.e10. doi:10.1016/j.molcel.2023.10.029 37977120

[36] Shen L , Qin W , Wu L , et al. Two novel presenilin‐1 mutations (I249L and P433S) in early onset Chinese Alzheimer's pedigrees and their functional characterization. Biochem Biophys Res Commun. 2019;516(1):264‐269. doi:10.1016/j.bbrc.2019.05.185 31235249

[37] Bagaria J , Bagyinszky E , An SSA . Genetics, functions, and clinical impact of presenilin‐1 (PSEN1) gene. Int J Mol Sci. 2022;23(18):10970. doi:10.3390/ijms231810970 36142879 PMC9504248

[38] Sato C , Takagi S , Tomita T , Iwatsubo T . The C‐terminal PAL motif and transmembrane domain 9 of presenilin 1 are involved in the formation of the catalytic pore of the γ‐secretase. J Neurosci.. 2008;28(24):6264‐6271. doi:10.1523/JNEUROSCI.1163-08.2008 18550769 PMC6670534

[39] Zhou R , Yang G , Guo X , Zhou Q , Lei J , Shi Y . Recognition of the amyloid precursor protein by human g‐secretase. Science. 2019;363(6428). doi:10.1126/science.aaw0930 30630874

[40] Devi G , Fotiou A , Jyrinji D , et al. Novel presenilin 1 mutations associated with early onset of dementia in a family with both early‐onset and late‐onset Alzheimer disease. Arch Neurol. 2000;57(10):1454‐1457. doi:10.1001/archneur.57.10.1454 11030797

[41] Jiao B , Tang B , Liu X , et al. Mutational analysis in early‐onset familial Alzheimer's disease in Mainland China. Neurobiol Aging. 2014;35(8):1957.e1‐1957.e19576. doi:10.1016/j.neurobiolaging.2014.02.014 24650794

[42] Rogaeva EA , Fafel KC , Song YQ , et al. Screening for PS1 mutations in a referral‐based series of AD cases: 21 novel mutations. Neurology. 2001;57(4):621‐625. doi:10.1212/WNL.57.4.621 11524469

[43] Taddei K , Kwok JBJ , Kril JJ , et al. Two novel presenilin‐1 mutations (Ser169Leu and Pro436Gln) associated with very early onset Alzheimerʼs disease. Neuroreport. 1998;9(14):3335‐3339. doi:10.1097/00001756-199810050-00034 9831473

[44] Potter R , Patterson BW , Elbert DL , et al. Increased in vivo amyloid‐b42 production, exchange, and loss in presenilin mutation carriers. Sci Transl Med. 2013;5(189). doi:10.1126/scitranslmed.3005615 PMC3838868 23761040

[45] Kakuda N , Takami M , Okochi M , Kasuga K , Ihara Y , Ikeuchi T . Switched Aβ43 generation in familial Alzheimer's disease with presenilin 1 mutation. Transl Psychiatry. 2021;11(1):558. doi:10.1038/s41398-021-01684-1 34728605 PMC8564532

