# Supplementary material for: The presenilin 1 mutation P436S causes familial Alzheimer's disease with elevated Aβ43 and atypical clinical manifestations
Source: Alzheimers Dement. 2024 Jun 2;20(7):4717–26. doi: 10.1002/alz.13904 (PMC11247678; doi:10.1002/alz.13904)
Supplement: Supplementary file 1 — Supporting information [file ALZ-20-4717-s001.docx]

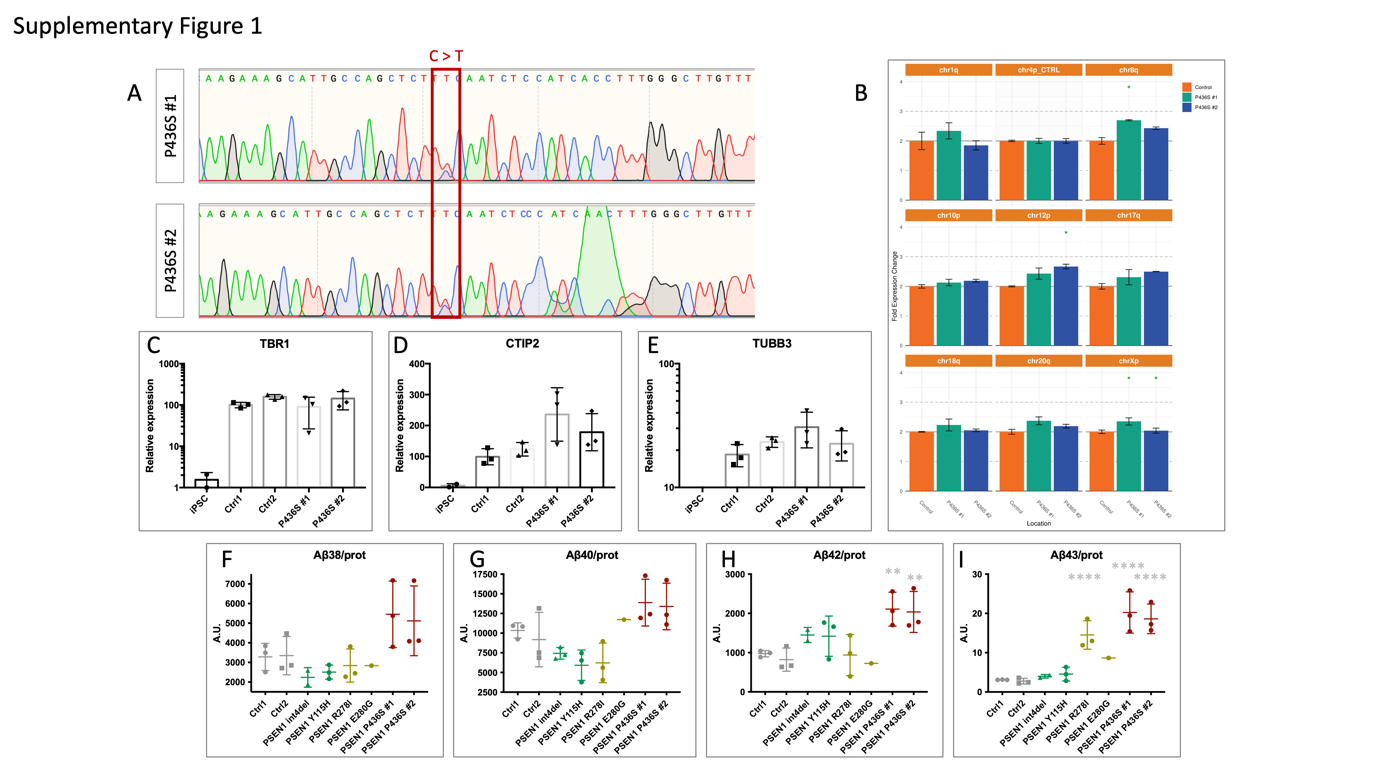


**Supplementary Figure 1. iPSC model characterisation.**

**A)** Sanger sequencing of *PSEN1* in iPSCs confirms the presence of the C>T mutation.

**B)** PCR-based karyotype analysis suggests a normal karyotype of the two P436S lines, green asterisks show possible amplifications that are not significant: P436S #1 has a possible amplification at Chr8q (copy number ~2.7, p=0.96); P436S #2 has a possible amplification at Chr12p (copy number ~2.67, p=0.099).

**C-E)** qPCR analysis of cortical marker expression in day 100 iPSC-derived neurons supports appropriate neuronal specification. TBR1, CTIP2 and TUBB3 are markers of deep layer cortical neurons, middle layer cortical neurons and pan-neuronal tubulin, respectively.

**F-I)** Aβ species quantification (pg/ml) normalised to the protein content of the corresponding neurons to estimate overall Aβ production. P436S mutations produce high levels of all species.

Significance represents one way ANOVA and Dunnett’s multiple comparisons test for each patient-derived line versus pooled controls, ** = p < 0.01, **** = p < 0.0001.


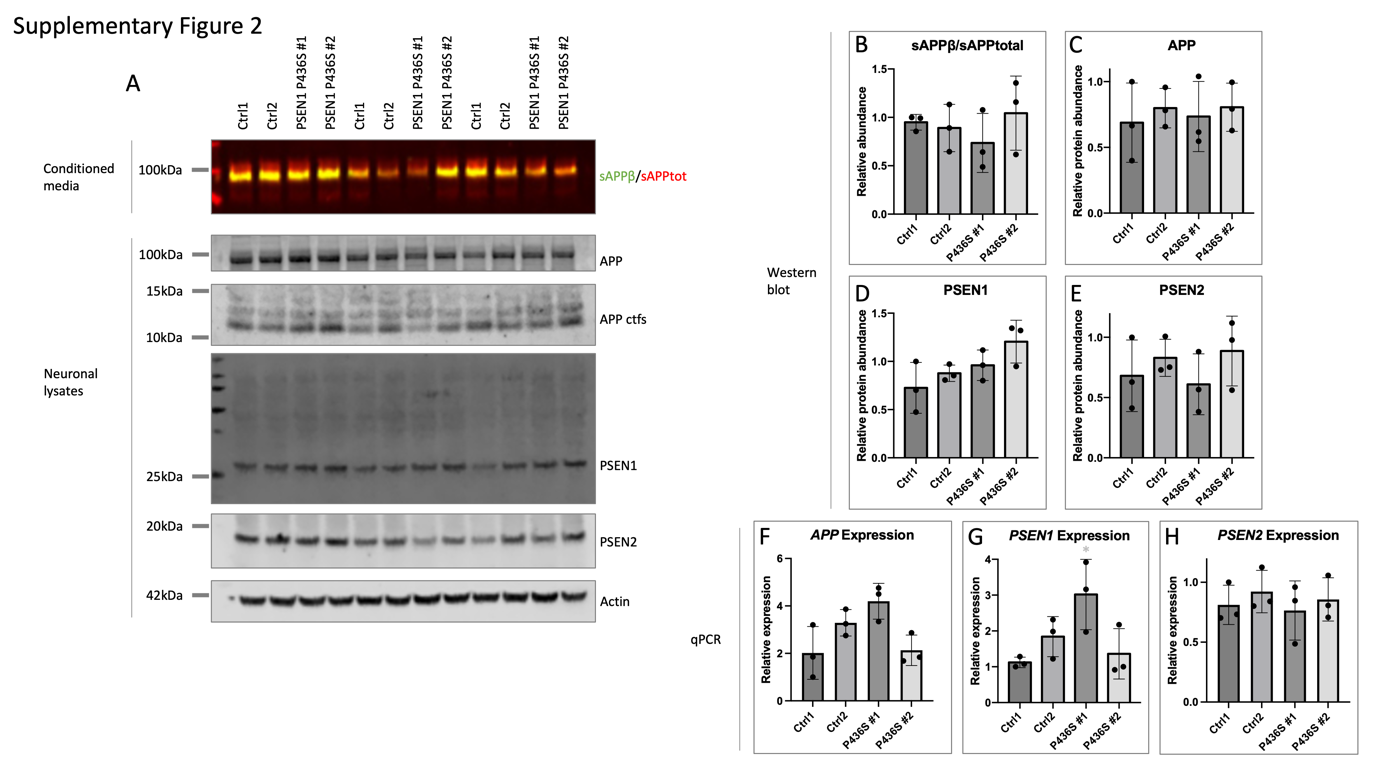


**Supplementary Figure 2. APP and PSEN1 expression in iPSC models.**

**A-E)** *PSEN1* P436S mutations do not affect amyloidogenic versus non-amyloidogenic processing, based on sAPPβ production levels, and do not affect APP, PSEN1 or PSEN2 protein level.

**F-H)** qPCR to show expression of *APP*, *PSEN1* and *PSEN2* in control and P436S neurons.

Protein and RNA analysis represent three independent neuronal inductions for each line.

Significance represents one way ANOVA and Dunnett’s multiple comparisons test for each patient-derived line versus pooled controls, * = p < 0.05.
